# Measurement of upper limb function in ALS: a structured review of current methods and future directions

**DOI:** 10.1007/s00415-022-11179-8

**Published:** 2022-05-25

**Authors:** C. D. Hayden, B. P. Murphy, O. Hardiman, D. Murray

**Affiliations:** 1 Trinity Centre for Biomedical Engineering, Trinity Biomedical Sciences Institute, Trinity College Dublin, Dublin 2, Ireland; 2 Department of Mechanical, Manufacturing and Biomedical Engineering, Trinity College Dublin, Dublin 2, Ireland; 3 Advanced Materials and Bioengineering Research Centre (AMBER), Trinity College Dublin, Dublin 2, Ireland; 4 Academic Unit of Neurology, Trinity Biomedical Sciences Institute, Trinity College Dublin, 152-160 Pearse St, Dublin 2, D02 R590 Ireland; 5 Neurocent Directorate, Beaumont Hospital, Beaumont, Dublin 9, Ireland

**Keywords:** ALS, Upper limb, Subjective, Technology, Outcome measurement

## Abstract

Measurement of upper limb function is critical for tracking clinical severity in amyotrophic lateral sclerosis (ALS). The Amyotrophic Lateral Sclerosis Rating Scale-revised (ALSFRS-r) is the primary outcome measure utilised in clinical trials and research in ALS. This scale is limited by floor and ceiling effects within subscales, such that clinically meaningful changes for subjects are often missed, impacting upon the evaluation of new drugs and treatments. Technology has the potential to provide sensitive, objective outcome measurement. This paper is a structured review of current methods and future trends in the measurement of upper limb function with a particular focus on ALS. Technologies that have the potential to radically change the upper limb measurement field and explore the limitations of current technological sensors and solutions in terms of costs and user suitability are discussed. The field is expanding but there remains an unmet need for simple, sensitive and clinically meaningful tests of upper limb function in ALS along with identifying consensus on the direction technology must take to meet this need.

## Introduction

Amyotrophic lateral sclerosis (ALS), also known as motor neurone disease (MND), is a rapidly progressive and ultimately fatal neurodegenerative disease characterized by degeneration of upper and lower motor neurons, with extra motor involvement increasingly recognised [1]. People with ALS experience muscle weakness and spasticity, which results in loss of limb function, respiratory impairment, loss of speech and swallow and in 20–50% cognitive and behavioural change [2]. In about two-thirds of cases, first symptoms appear in the limbs [3], which manifest in problems such as inability to raise the arms, loss of hand dexterity, foot drop, and difficulty walking [4]. A recent study [5] on disease progression reported that symptom development in ALS appeared to be an organised process, with onset in the arm occurring more than bulbar and leg onset, respectively. Among arm-onset patients, involvement of the contralateral arm developed significantly faster compared to other sites.

Currently, there are two drugs approved for ALS: Riluzole, which provides a modest benefit of slowing disease progression; and Edaravone, which has shown limited efficacy in a highly selected cohort of patients [6]. The primary endpoint in the trials for these drugs and in the majority of ALS clinical trials to date has been the Amyotrophic Lateral Sclerosis Rating Scale Revised (ALSFRS-r) [7]. This multi-item ordinal scale relies on reproducible scoring by a trained rater in consultation with the patient, assigning a level of functioning from zero to four for each of twelve domains. It includes specific upper limb items: handwriting, cutting food and handling cutlery and dressing and washing. However, problems with construct validity have been reported and the slope shows a non-linear longitudinal decline [8, 9]. Moreover, analysis of the subgroups within the ALSFRS-r demonstrates floor and ceiling effects, which limit sensitivity and significantly increases the risk of failure to identify a real effect of an intervention under investigation [9, 10].

The measurement of patient outcomes could be improved using additional technology-assisted outcomes [11], such as Inertial Measurement Units (IMUs), activity monitors and motion analysis systems. Such technologies, if widely used, have the potential to address the subjectivity of current measures such as the ALSFRS-r. Additionally, the integration of technology in assessment provides opportunities for remote monitoring and remote data collection in clinical trials [12].

The aim of this paper is to present a structured review of the literature pertaining to both traditional, low tech, measurement tools currently used for assessment of upper limb function and hand dexterity with a specific focus on their application to ALS; and novel technology-enabled devices that will in future provide quantitative measurement of upper limb function and dexterity. Improved measurement of motor function of the upper limb confers an increased power to detect changes for novel therapeutic approaches. Challenges and opportunities in devising and implementing technology are discussed.

## Methodology

The authors reviewed the literature available on Google Scholar, PubMed, Scopus and general search engines. This structured review includes representative papers in each of the traditional and technology sections as defined by the authors. The following main keywords were used to identify papers of interest which were then assessed by the authors: (1) ALS, amyotrophic lateral sclerosis, MND, motor neurone disease; (2) upper limb, finger tapping test; (3) medical device; (4) neurology, neuromuscular diseases. Inclusion criteria were not limited to ALS focused devices. Any novel device that focused on upper limb impairment was included if there was not a specific ALS equivalent. Exclusion criteria was as follows: posters, technology-based devices developed for healthy participants and multiple papers that used the same technology-based sensors. From this, a representative sample of 43 traditional upper limb measurement papers and 47 technology-based papers were chosen that provide a structured review of the overall field.

## Traditional upper limb measurement

Forty-three papers were reviewed which employed traditional upper limb measurement. Assessment of upper limb measurement purports to examine both gross and fine motor control. In ALS this is currently assessed by three questions of the ALSFRS-r, which score handwriting, using utensils or feeding tube fastenings and managing dressing and hygiene. Limitations on detecting impairment resulting from hand dominance versus the affected limb have been recognised, as well as the inability to accommodate for cultural differences [13, 14]. A limited number of trials incorporate objective outcomes by addition of objective measures such as manually picking up objects. Traditional measurement tools include questionnaires, objective functional grading scales such as the Action Research arm Test (ARAT) [15] and Motor Assessment Scale (MAS) [16], and objective tests of impairment including dynamometry for strength measurement, pinch and grip strength testing, gross motors tests such as the box and block test and fine motor tests like the finger tapping test and nine-hole peg test (NHPT). These traditional tests are outlined in Table 1.

**Table 1:** Review of the subjective paper-based questionnaires that focus on upper limb function measurement

Questionnaires	Condition	Method	Upper limb functioning assessed	Limitations
Subjective scales-clinician rated scales				
ALSFRS-r Validated rating instrument for monitoring ALS disease and progression [7]	ALS	12 functional questions. Responses rated 0–4. Scores summed to give result between 0 and 48	Three upper limb focused questions relating to handwriting, using utensils and dressing	Not sensitive to small changes Influenced by handedness Non-linear decline
DASH (Disabilities of the Arm, Shoulder and Hand) General purpose measure for cross section of conditions [17, 18]	General	30- Item questionnaire; examines patients’ ability to perform certain upper extremity activities. Scores rated from 1 to 5. Scoring range from 30 to 120 which is then scaled between 0 and 100	Subjective questions relating to functional tasks such as ability to wash or use knife	Unidimensional Region specific, not joint specific Score may be influenced by lower extremity disability
Subjective scales–self (patient) rated				
Upper Extremity Functional Index (UEFI) Used to assess functional impairment [19]	General	Self-reported questionnaire. 20 or 15 item versions. Responses rated from 0 to 4. Scores are then summed for total. 15 item version scaled to between 0 and 100	Functional questions include tying shoelaces, dressing, feeding and tasks such as opening a jar or lifting	Large 9-point change required for meaningful change Self-reported
Patient-Specific Functional Scale (PSFS) Applicable for large range of clinical presentations [20, 21]	General	Self-reported outcome measure for patients with back, neck, knee, and upper extremity problems. Patients select 5 activities they are having difficulty performing. Rated on 11-point scale (0–10). Final score = Sum of the activity scores/Number of activities registered	Patient focused—activities focused on upper limb movement if that is the affected area	Self-reported Comparison between patients or groups of patients limited due to patient focused nature
ABILHAND Questionnaire Self-reported assessment measures perceived difficulty [22, 23]	General	Self-administrated questionnaire. Various versions. Original 56 item version, 4 level scale Also 23 item version with 3 level scale	Functional questions such as writing, cutting, and dressing	Self-reported Only suitable for patients without cognitive defects
Michigan Hand Questionnaire (MHQ) General measure of hand outcomes [24, 25]	General	Patient rated questionnaire. 37 items divided into 6 categories. 5 level scale. Each category is summed individually and scaled to give values between 0 and 100	Focused only on hand outcomes. Sections on daily living, function, work and pain. Also includes section on aesthetics	Self-reported Relatively time–consuming to complete (mean approx. 10 min)
Arm Activity Measure (ArmA) Measure of difficulty in passive and active functions UL daily tasks [26]	General, with emphasis on spasticity	Current version is eight item passive function subscale and a 13-item active function subscale. Responses rate from 0 to 4. Subscales summed separately and not combined	All questions in both sections focus on arm specific tasks such as cutting fingernails, eating and drinking	Self-reported Unidimensional—passive and active questions are separate scores
Clinician rated observational scales				
ARAT (Action Research Arm Test) General outcome measure reliable in populations such as stroke [27]and multiple sclerosis [28]	General	19 items across 4 areas; grasp, rip, pinch and gross movement Scale is set from 0 -3. Total score ranges from 0 -57	Four subscales (grasp, grip, pinch, and gross movement) Subjects asked to lift grip objects such as paper, blocks and balls	Significant floor and ceiling effect Unidimensional
Movement Disorder Society-Sponsored Unified Parkinson’s disease rating scale (MDS—UPDRS) Main rating tool used for PD, developed to improve old version [29, 30]	Parkinson’s Disease	4 sections. 50 item scale. Scores rated from 0 to 4 and summed together to get total	Section 2 has self-evaluating questions on handwriting, cutting food, using utensils etc Section 3 focuses on evaluating motor function; specific question on finger taps and hand movements	No screening questions for non-motor aspects Not free to use outside of individual/personal use Approximately 30 min to complete
Barthel Scale/Index (BI) Intended to assess and monitor disability over time [31, 32]	General	Ordinal scale—measure performance in activities of daily living. Most recent version has 10 activities rated from 0 to 2. Scores multiplied by 5 to get number out of 100	Sections on feeding, grooming and dressing	Ceiling effect—poor ability to detect change in highly functional individuals Not recommended to be used alone for predicting outcomes – low sensitivity
Functional Independence Measure (FIM) Intended as improved Barthel Scale and measure of disability [33, 34]	General	18-Item measurement tool divided into 6 sections, intended for patients with functional mobility impairments. Divided into two domains: motor and cognition. Scores range from 1 to 7 and are summed to get total range (18–126)	Sections on feeding, grooming and upper body dressing	Unidimensional—but validity of using score to represent single value is still debated
Motor Activity Log (MAL) Scripted questionnaire to examine impaired arm use outside laboratory tests [35]	Stroke	Subjective measure of individual’s functional upper limb performance. Versions range from 12 to 30 questions. Responses rated 0–5. Mean score calculated by adding scores for each scale and dividing by number of questions asked	Questions asked include ability to write on paper, use fork or spoon, put on clothes and removing item from drawer	Experimenter bias Patient recall ability Relies on self-ratings
Motor Assessment Scale (MAS) Assesses functional tasks [36]	Stroke	8-Item scale assessed using a 7-point hierarchy (0–6 score). Items scores (excluding general tonus item, which uses different scoring criteria) add to a max of 48	Sections on upper arm function, hand movement and advanced hand activities	General tonus section difficult to assess reliably Problems in scoring hierarchy associated with advanced hand activities
Wolf Motor Function Test Focused on upper extremity performance [37]	Stroke	17-Item test that utilizes equipment. Contains 3 parts focusing on functional tasks, strength measurement and movement quality 6-point scale (0–5). Lower scores indicate lower functioning levels	Items have questions on picking up paper clip, picking up pencil and using pincer grip	Not quick to administer (30 min +)
Rivermead Motor Assessment–arm section Measures functional mobility [38]	Stroke	33-Item scale that utilizes equipment with three subscales. Response either 0 or 1. Specific order to questions that presumes that each subsequent item is of a more difficult nature. Each subscale scored by summing the points allocated for all items within that subscale	15 questions in Arm section of scale. Tests involve picking up sheet of paper from table or cutting putty into pieces with knife and fork	Floor effect Limited score range
Canadian Neurological Scale (CNS) Designed to measure mentation and motor function [39]	Stroke	8-Item scale. Two sections on motor function evaluation depending on patient condition. Scores from each section summed to give max section score of 11.5	Two questions that apply force to elbows when lifted to shoulder height or apply pressure to back of hand. Movement rated 0–1.5 in blocks of 0.5	Only focuses on limb weakness

At present, there is no consensus between specific questions and the rating system used. The subjective nature of these questionnaires has led to the incorporation of additional objective instruments, as is the case with the ARAT and Jebsen Hand Function Test. These hybrid evaluation tools include sections on tasks related to fine motor control which can be objectively recorded, usually with a stopwatch. However, all inherent subjective biases remain, for example, a delay in a tester starting a stopwatch. Moreover, there has been no cross validation with disease specific scales such as the ALSFRS-r. To the authors’ knowledge, only the NHPT has seen limited use in ALS-specific studies [40].

Due to the subjective nature of the neurological questionnaires, several performance-based tests have been included as part of clinical evaluation (see Table 2). A commonly used instrument is the nine-hole peg test (NHPT), which measures hand dexterity. This has been validated in all age groups, has high interrater validity and is sensitive to patients with neuromuscular or musculoskeletal conditions [41]. It is commercially available, quick, easy to administer and has a minimal ceiling effect. Limitations include the complexity of the task, which can be challenging for patients with cognitive impairment, and the early floor effect for moderate to severe hand impairment, where some useful function of the hand remains but the test cannot be completed.

**Table 2:** Review of the most popular functional tests that accompany the paper–based questionnaires in an attempt to provide an objective score

		Method	Advantages	Disadvantages
Test				
Nine-hole peg		9 pegs in container – participant places them in holes as fast as possible, then removes them Timed with stopwatch	Easy to administer Good reliability and validity [42]	Practice effects [43]
Perdue peg board		Rectangular board–2 sets of 25 holes running vertically and 4 cups at the top Pegs placed in cup on side being tested, participant places pegs as fast as possible Number of pegs placed in 30 s scored	Short, easy to administer and score [44] 5 scores—right, left, both hands, total of those, and assembly	Limited to patient cohort with relative high degree of fine motor and cognitive skills
O’Connor finger dexterity test		Two versions: 100 pins placed in 100 holes using hands or 300 pins placed in 100 holes using tweezers Timed with stopwatch	No training required Easy to use [45]	Much longer compared to similar tests Only returns one score
Minnesota manual dexterity test		Board with 60 holes and 60 blocks 2 subsets: placing blocks in hole one by one and turning the blocks over Scoring is time taken	Good validity and test–retest reliability [46]	Only power grip information gathered
Box and block test		Box with partition–150 blocks on one side. Blocks moved from one side to the other, one at a time Score is number of blocks moved in 60 s	Quick, easy to administer Excellent validity with questionnaires [47]	More expensive than peg tests Test requires rapid movement
Hand dynamometer		Grip Strength Test–usually an accompaniment to fine motor test Participant grips dynamometer as hard as possible	Portable Large amount of normative data available [48]	Stress on weak joints – heavy Affected by hand size Repeatability issues: hand position is different between tests
Jebsen hand function test (JHFT/JTT)		Developed to provide objective measure of fine/gross motor function [49] Objective measure of gross motor hand function using simulated activities of daily living (ADL). 7 subsets. Score is sum of time taken for each test, rounded to nearest second	Portable Standardised instructions	Practice effects Sections on picking up small common objects such as coins and moving large empty/weighted cans respectively

The Finger Tapping Test (FTT) is one of the most widely used measures of motor function in neurological practice [50, 51]. It involves tapping the index finger against the thumb rapidly while the clinician judges whether the movement is normal or abnormal by visually evaluating amplitude, frequency and accuracy. Visual grading is subjective and for non-expert evaluators, is insensitive to small but meaningful changes. There are currently two main methods used to evaluate the FTT; tip of index finger to tip of thumb or tip of index finger to distal crease of thumb with the distal crease of the thumb suggested as a more sensitive measure [52]. Commercial objective versions of the FTT are limited to simple tapping devices, as these are integral to the Halstead-Reitan Neuropsychological Battery (HRNB), a widely used battery that contains a finger tapping test. This instrument uses a tapping lever mounted with a key-driven mechanical counter [53]. Other devices include the light beam finger tapping test [54, 55], which has limited utility as it is cumbersome and has limited benefits when compared with the current visual assessment used by expert clinicians.

## Technology based solutions for upper limb measurement

**Fig. 1:**
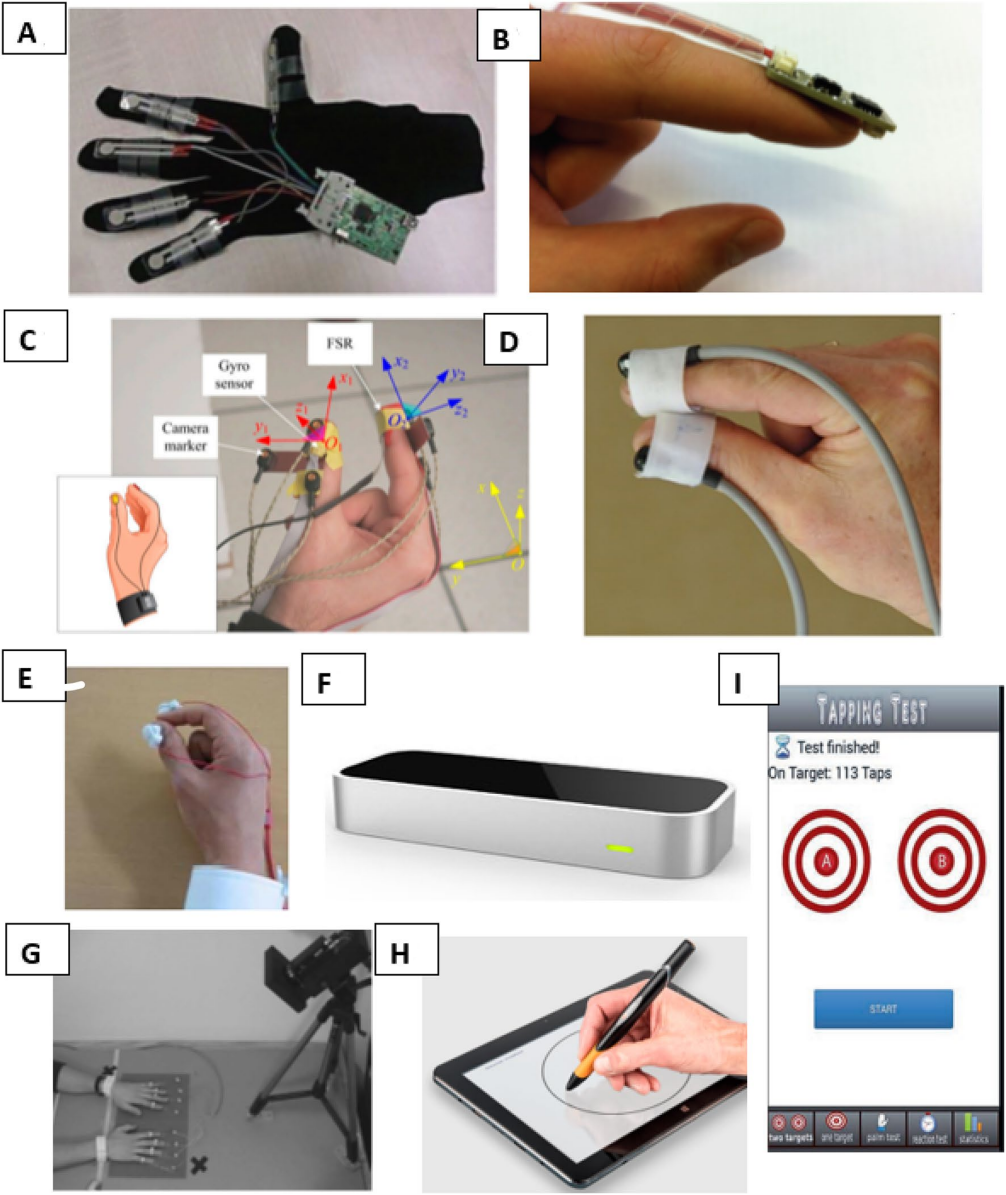
**A** Typical example of a glove-based device [56], **B** accelerometers can be attached to various positions on the hand and wrist to capture movement in terms of acceleration, seen here placed on index finger [59], **C** gyroscope sensors measure orientation and angular velocity, can be positioned anywhere, seen here with device that fits on thumb and index finger [78], **D** image of the inertial measurement unit (IMU) developed PD-Monitor, a commercial PD device that focuses on a finger tapping test [66], **E** magnetometers offer a counterpoint to accelerometer and gyroscopes but are not used much on their own, image shows a device that relies on two magnetometers [65], **F** Leap Motion Controller (Leap Motion Inc., San Francisco, USA.), a commercial system that detects the motion and portion of the hand using infrared (IR) sensors, **G** A 3D Marker-based camera setup where position is determined through the use of reflective markers [71], **H** a digital pen (Manus Neurodynamica Ltd.) that aims to quantify handwriting, along with tablet stylus’ they are bracketed into mobile application devices [79], **I** example of a mobile app interface designed to measure a tapping test [80]

There has been a substantial increase in the number of novel sensor devices available which have been broadly classified into 4 categories, direct measurement, indirect measurement, keyboard surrogates and mobile applications. These classifications have been synthesised by the authors to distinguish the main differences in measurement methodology. Table 3 provides a summary of the main devices in these four categories including mechanical and clinical advantages. Forty-five papers were found that evaluate these different technology categories. Figure 1 displays a selection of images of a selection of the technology-based sensors.

**Table 3:** Technology-based sensors that have been used to objectively measure upper limb function

Device	Category	Examples	Mechanical		Clinical	
			(+)	(−)	(+)	(−)
Glove based	Direct measurement	[56–58]	Quick setup, detailed measurement of joints possible	Obtrusive	Easy setup	Hygiene issues, not suitable for all patients
Accelerometer	Direct measurement	[59–61]	Measures linear acceleration, small, cheap	Only measures linear movement, noise, gravitational artefacts	Easy setup, hygienic, potential for remote monitoring,	Interfere with normal finger tapping motion, placement, requires training
Gyroscope	Direct measurement	[62–64]	Measures orientation and angular velocity Lightweight	Artifacts		
Magnetometer	Direct measurement	[65]	Measures magnetic field change in x, y, z directions Lightweight, accurate No artifacts	Errors when coil orientation changed, possibly sensitive to presence of magnetic/ferromagnetic objects		
IMU	Direct measurement	[66–68]	Detailed measurement of joints	Accumulated error, noise, gravitational artefacts		
Optical w. markers	Indirect measurement	[69–71]	Accurate–markers provide exact position	Occlusion, expensive, stationary	Hygienic–no patient contact	Not bedside friendly
Optical n. markers	Indirect measurement	[72]	Contactless, cheap	Occlusion, limited accuracy	No patient contact	Not bedside friendly
Mobile apps	Mobile Applications	[73, 74]	May include additional tools such as tablet stylus/digital pen outside phone, Remote monitoring	Software limitation, unable to monitor finger movement	Remote monitoring	Require technology
Keyboard surrogate	Keyboard surrogate	[75–77]	Cheap, easy to use	Can only record finger motion when touching key, limited	Easy to use	Problematic to clean

Direct measurement devices encompass accelerometers, gyroscopes, magnetometers, and inertial measurement units (IMUs). Accelerometer devices which are placed on the index finger and record the acceleration as a finger tap have been developed [59, 60, 81]. Gyroscopes have been used to measure bradykinesia or tremors in Parkinson’s disease (PD) patients [63, 78, 82]. Inertial measurement units (IMUs) combine the input from several different sensors to give a more accurate output of movement. A range of studies [66–68, 83, 84] have examined different IMUs for use in hand and finger tracking, most associated with the finger tapping test.

Glove-based systems provide quantitative analysis of hand function, which can be used to guide rehabilitation and improve the patient’s recovery, [57, 85–88]. However, these devices interfere with normal movement as they cover the hand and pose difficulties with respect to hygiene. Although each sensor has strengths (Table 3), a common issue most with most direct measurement devices is noise, and sensor placement can be extremely varied which limits consensus between researchers.

Indirect measurement devices focus on optical sensor systems that offer an alternative to physical devices placed on a subject’s hand or fingers. There are a number of commercially available systems, such as Vicon (Vicon, Oxford, UK), which use a high-resolution camera setup and strategically placed reflective markers placed on the body. Motion capture systems are more accurate when markers are placed on the participant’s body and used for positioning. Most other marker-based optical systems use either passive or active markers to determine position, but some used a combined camera-based approach with IMUs used as the markers substitute [70]. Systems that record motion capture without the use of markers based on algorithms and pattern recognition. Most systems are expensive and unvalidated in a clinical setting. The Microsoft Kinect and Leap Motion Controller (Leap Motion Inc., San Francisco, USA)) are relatively inexpensive motion capture-based systems. The Kinect has been used [89, 90] to examine reachable workspace as a potential outcome measure in neurological conditions. This system correlated findings with gross motor sub scores of the ALSFRS-r; however, currently available systems are limited in resolution when measuring fine motor movements [91]. The size and space needed for most of the systems also render them unsuitable to clinical settings.

Keyboard typing negates the need for additional sensors and the equipment is readily available. Combinations of keyboard and sensors have been used to quantify upper limb impairment in ALS patients, and to determining a sensitive marker that could be used to monitor disease progression. Other methods such as tapping specific keys [92], calculating an interkeystroke interval (IKI) parameter [75], and determining motor speed from tapping a gaming mouse [93] have also been developed. Although this type of measurement is easy to set up, it is limited as data can only be gathered when tapping the key.

Mobile applications allow for remote monitoring and provide feedback on disease progression. These offer remote monitoring combined with objective testing. Due to the advances in smartphone technology, most phones are now equipped with accelerometers and gyroscopes that can be utilised to provide an accuracy similar to laboratory settings, depending on the measurement aims. Smartphone screens are sensitive to touch and also offer an alternative to the keyboard systems. Most mobile applications use a modified version of the Finger Tapping Test but similar to the keyboard devices, they are limited in their ability to record with data gathered mostly surrounding index finger amplitude and velocity [94–101]. Berry et al. [102] have reported on the benefits of using a mobile app for a self-administered ALSFRS-r, PD applications have been developed that gather hand function information in PD. There is a further additional to this category with the development of other novel tools such as digital pens, for example, the NeuroMotor Pen (Manus Neurodynamica Ltd), that aim to quantify handwriting ability. These are used in conjunction with mobile platforms with the aim of easily integrating them into current commercially available devices (i.e., iPad (Apple Inc.)).

## Discussion and conclusion

This review summarised the current literature in relation to the measurement of upper limb function in ALS and included forty-three papers on traditional and forty-five on novel technology-based assessment solutions. There is a paucity of ALS-specific research in this area and the majority of the studies discussed are not ALS specific, as most of the scales and measurement devices developed have focused on other neurological conditions such as PD. Nonetheless, the identified strengths and limitatio ns of these scales and devices and the learnings from these studies are applicable to ALS. The advantages and disadvantages outlined in Tables 1, 2, 3 are universal across neurological conditions and highlight an unmet need for novel, technology-based solutions for assessment of upper limb function.

Sensors such as accelerometers or motion capture systems are cheap, and available with software that supports their use in clinical settings. However, all current systems have limitations, and there is no clear leader in the field. While integration with currently validated questionnaires is important, care must be taken not to limit the potential of an objective sensor by tying it too closely to the subjective questionnaires.

**Fig. 2:**
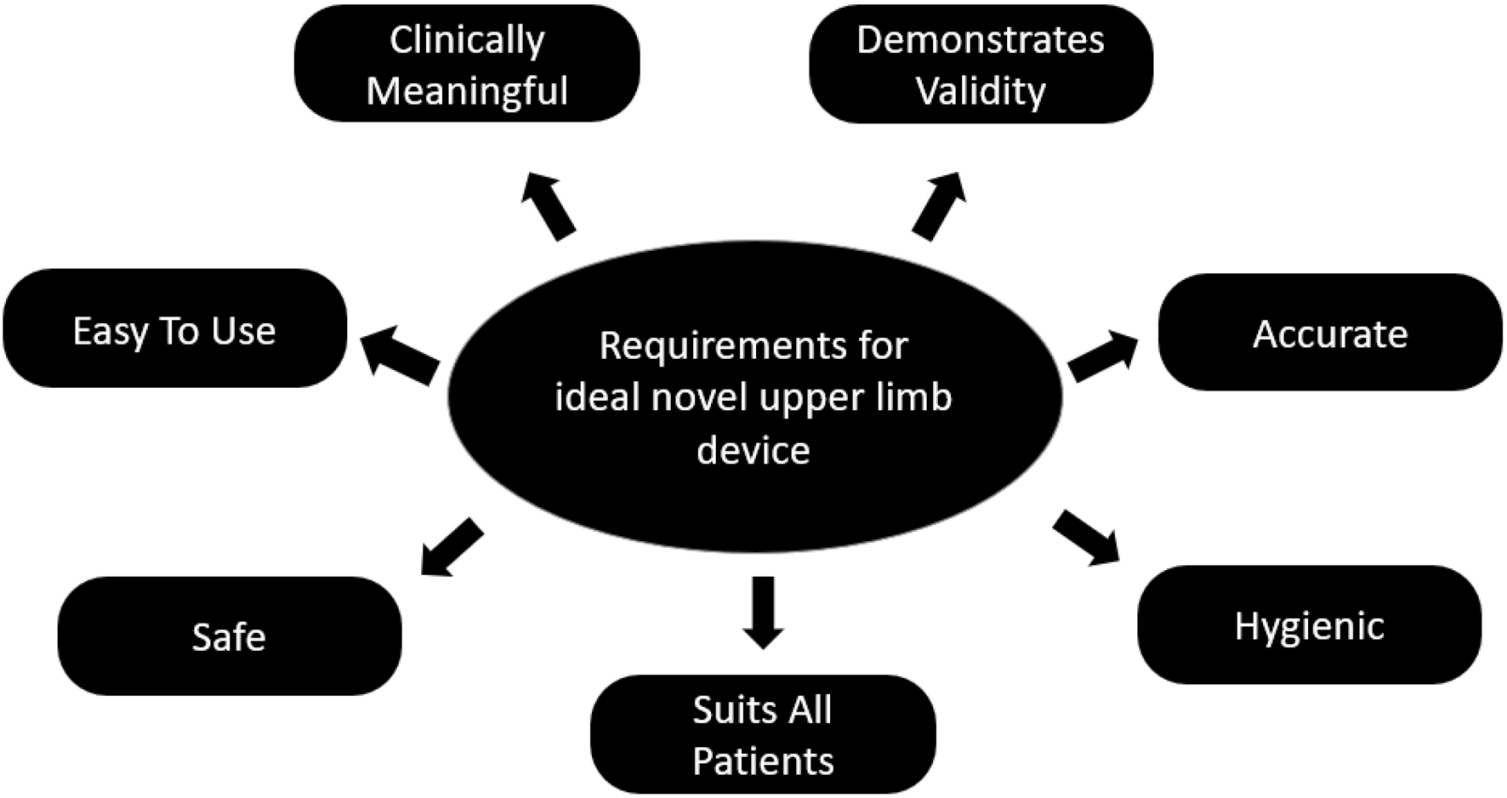
Image highlighting the key minimum requirements that an ideal modern sensor device should have

For technology to be effectively used for measurement of hand function or dexterity, it must provide an objective measure of hand function, which is clinically meaningful and sensitive to small but meaningful changes and designed with the patient and clinician in mind (Fig. 2). The rapidly progressive nature of symptoms in ALS provides an additional challenge as assessment tools must be suitable for frequent use and ideally for remote monitoring. Many currently available novel measurements are limited by issues such as cost or complexity of assessment setup and are not amenable to frequent use or suitable for remote monitoring. Simple and widely used measurement tools such as hand grip dynamometry are limited in ALS by rapidly progressive weakness and presence of a floor effect, while some meaningful hand function (e.g., tapping a tablet screen) is preserved.

Data privacy and CE marking of novel devices or algorithms must also be taken into consideration [103]. Adoption of any new device is dependent on the strategies surrounding the CE mark and operational aspects, which reflect decisions that need to be taken early in the development of a device. Clinicians must be satisfied a novel device will give precise, reliable and continuous information about patient limb position and function [104] especially if the information will be used to inform clinical decisions. A thoughtf ully designed sensitive device has the potential to provide enhanced information, which in turn improves the efficiency of clinical trial evaluations [105].

The benefits of technology are clearly recognized. In ALS, the challenge is to develop assessment devices that will adequately address the current limitations of current measurement instruments such as the ALSFRS-R in a reproducible, user-friendly and inexpensive manner. While no currently available device has met all of the necessary criteria to ensure universal acceptance in clinical practice (Fig. 2), there is clearly a demand for technological innovation which will be best achieved by ongoing collaboration between bioengineers and expert clinical professionals.
